# Supplementary material for: Identification of a βCD-Based Hyper-Branched Negatively Charged Polymer as HSV-2 and RSV Inhibitor
Source: Int J Mol Sci. 2022 Aug 4;23(15):8701. doi: 10.3390/ijms23158701 (PMC9369026; doi:10.3390/ijms23158701)
Supplement: Supplementary file 1 [file ijms-23-08701-s001.zip › ijms-1852034-supplementary.pdf]

Identification of a βCD-Based Hyper-Branched Negatively Charged Polymer as HSV-2 and RSV Inhibitor

Rachele Francese [1#], Claudio Cecone [2#], Matteo Costantino [1], Gjylije Hoti [2], Pierangiola Bracco [2], David Lembo [1*], Francesco Trotta [2*]

[1] *Laboratory of Molecular Virology and Antiviral Research, Department of Clinical and Biological Sciences, University of Turin, Italy*

[2] *Department of Chemistry, Nis Interdepartmental Centre, University of Turin, Via P. Giuria 7, Turin, 10125, Italy*

#RF and CC: these authors equally contributed to the work

*Corresponding authors

Supplementary Material

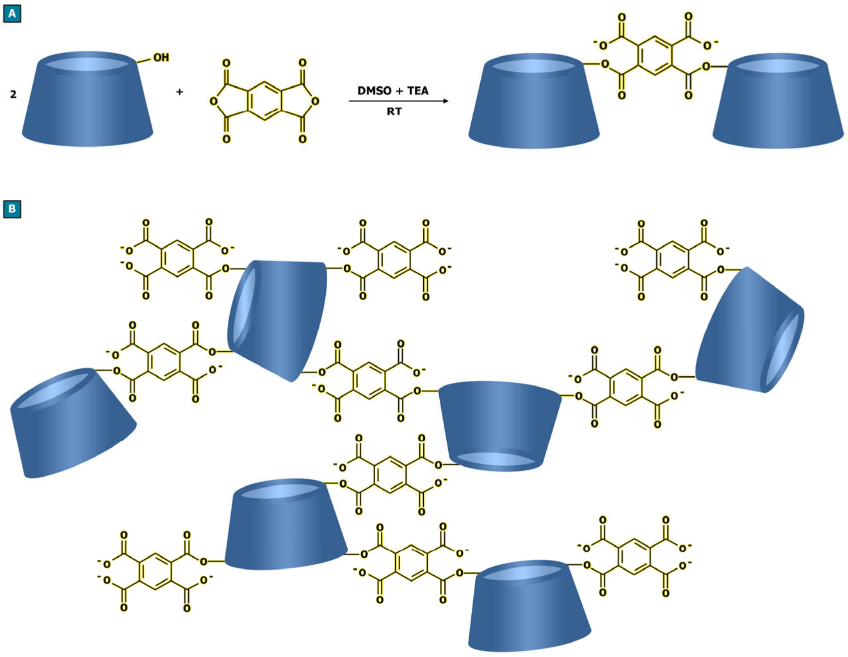

Figure S1. (A) Reaction occurring between βCD and PMDA, (B) P_PMDA proposed structure.

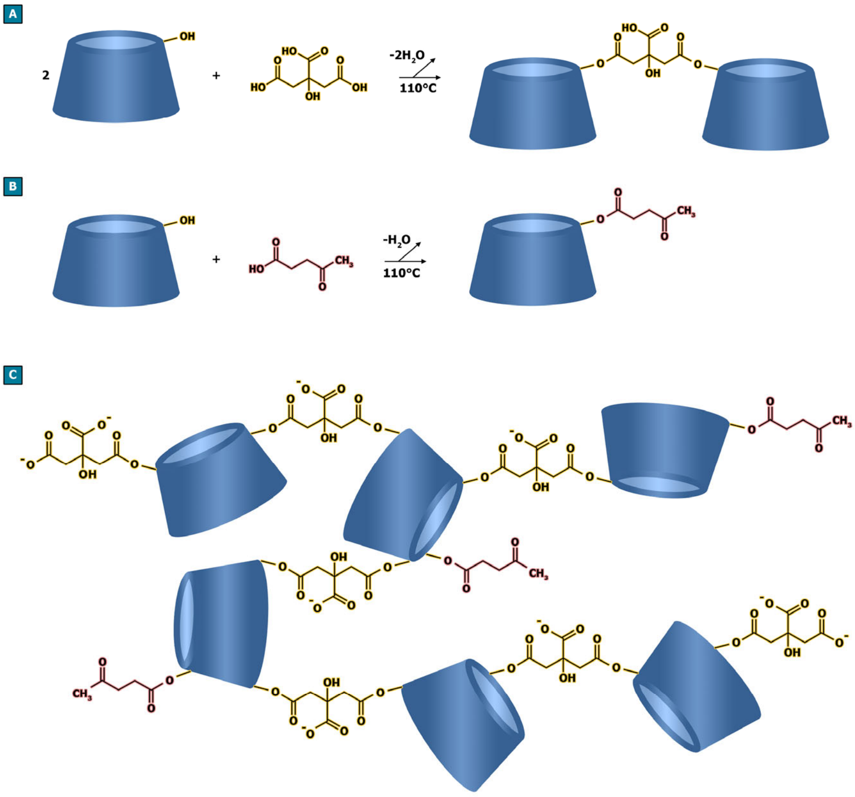

Figure S2. (A) Reaction occurring between βCD and CA, (B) Reaction occurring between βCD and LA, (C) P_CA_LA proposed structure.

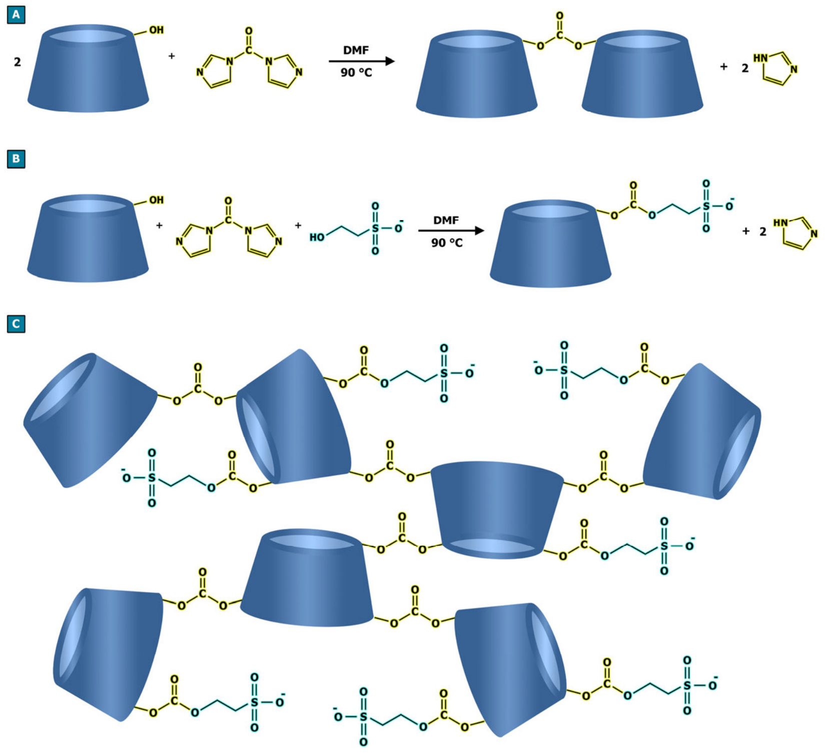

Figure S3. (A) Reaction occurring between βCD and CDI, (B) Reaction occurring between βCD, CDI, and ISE, (C) P_CDI_ISE proposed structure.

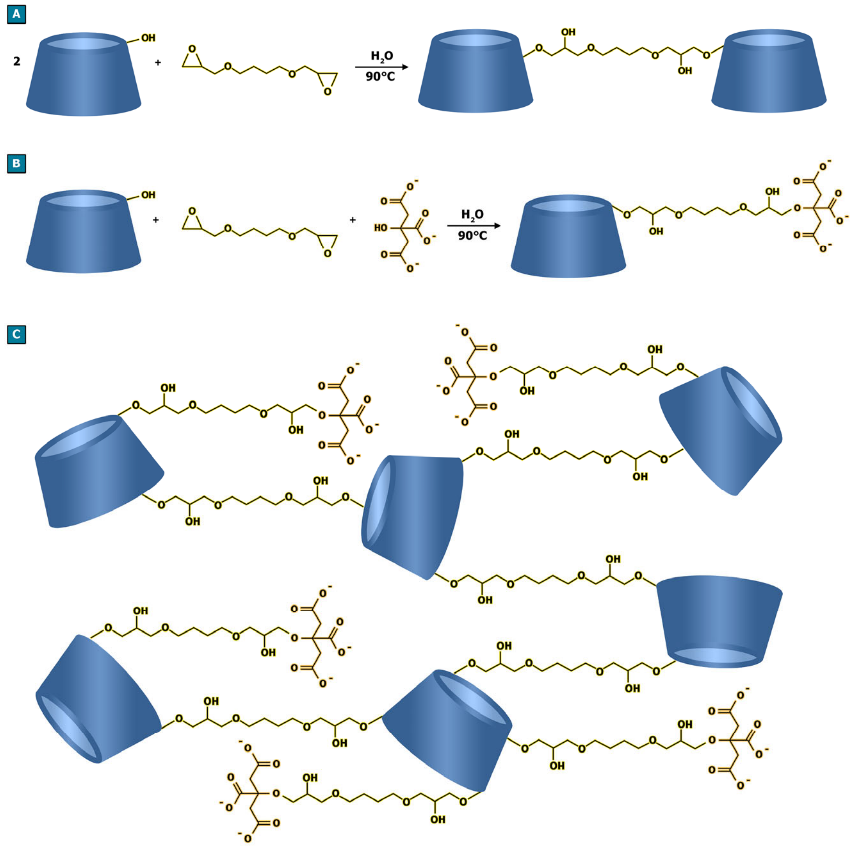

Figure S4. (A) Reaction occurring between βCD and BDE, (B) Reaction occurring between βCD, BDE, and CIT, (C) P_BDE_CIT proposed structure.

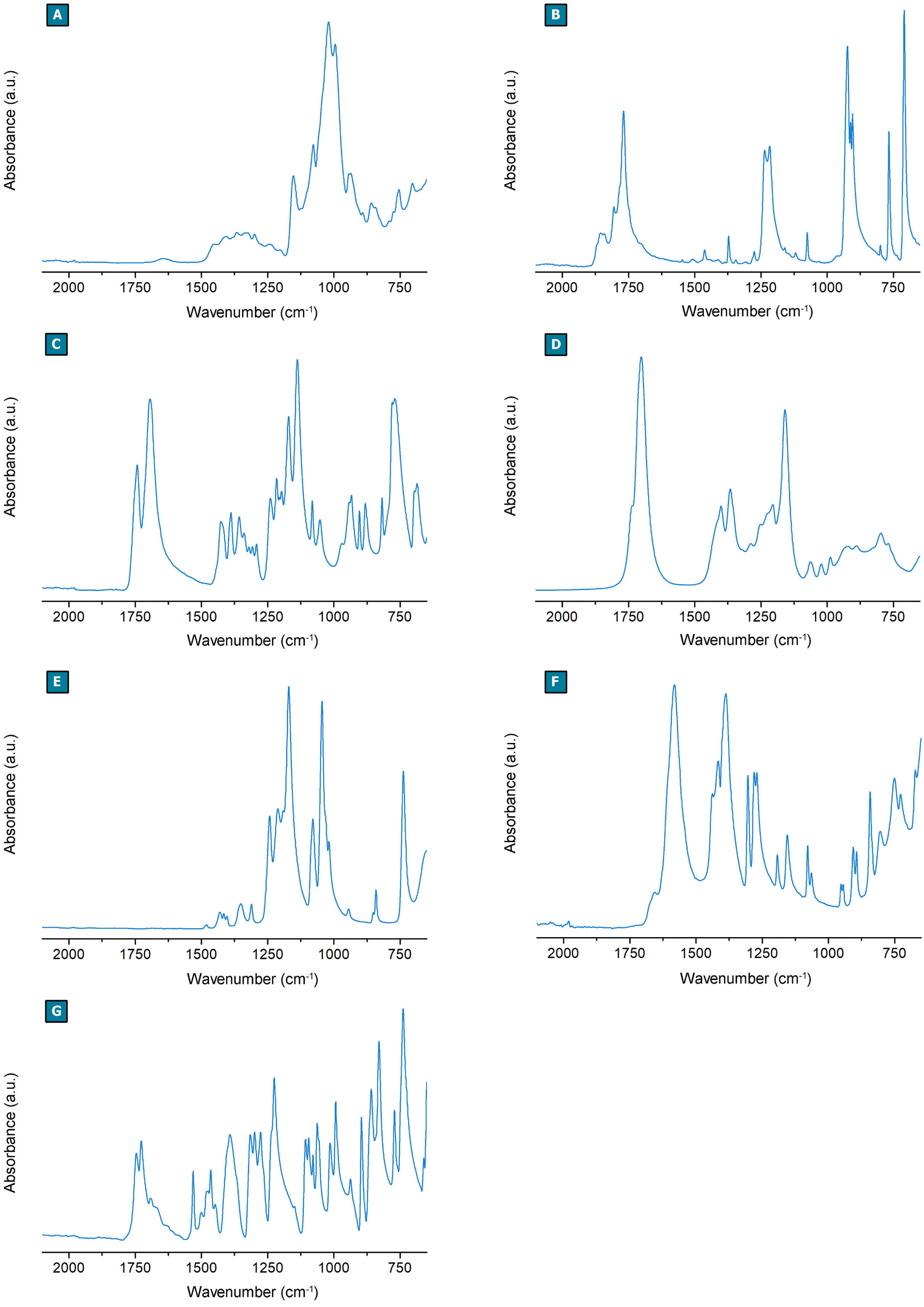

Figure S5. FTIR-ATR spectra of (A) βCD, (B) PMDA, (C) CA, (D) LA, (E) ISE, (F) CIT, (G) CDI.

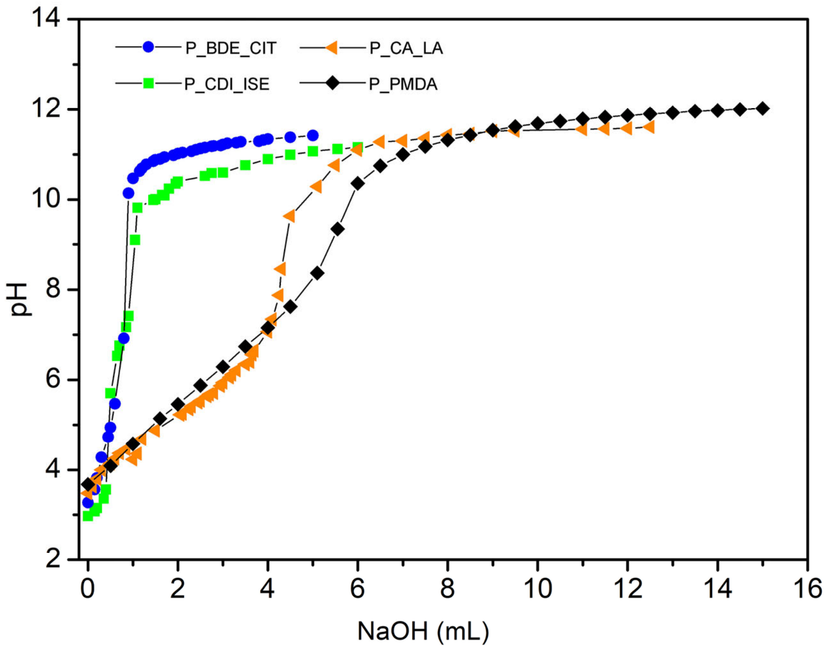

Figure S6. Potentiometric titration curves.

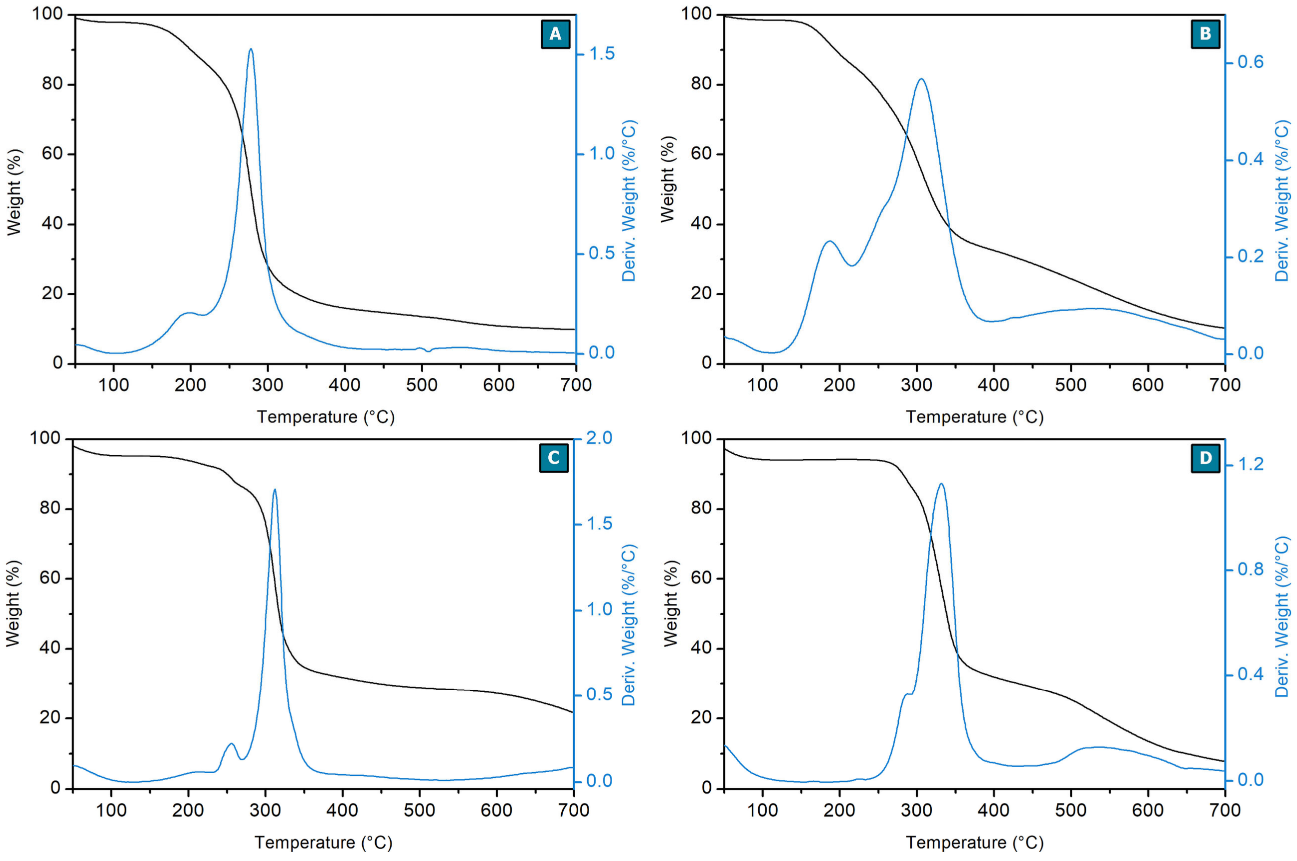

Figure S7. TGA and DTGA of (A) P_PMDA, (B) P_CA_LA (C) P_CDI_ISE, and (D) P_BDE_CIT.

Table S1. Raw data of P_PMDA and P_PMDA$_{50}$ virucidal activities

	Sample	Titer (PFU/mL)	*R
**RSV**	Untreated	257178	
	P_PMDA (500 µg/mL)	18471	1.1
	P_PMDA (300 µg/mL)	43170	0.8
	P_PMDA (100 µg/mL)	107325	0.4
	P_PMDA$_{50}$ (500 µg/mL)	30101	0.9
	P_PMDA$_{50}$ (300 µg/mL)	43486	0.8
	P_PMDA$_{50}$ (100 µg/mL)	200138	0.1
**HSV-2**	Untreated	897075	
	P_PMDA (1.5 µg/mL)	599075	0.2
	P_PMDA (15 µg/mL)	315150	0.5
	P_PMDA (100 µg/mL)	330480	0.4
	P_PMDA$_{50}$ (1.5 µg/mL)	493514	0.3
	P_PMDA$_{50}$ (15 µg/mL)	203016	0.6

***Logarithm of inactivation R** = Log (Untreated sample) – Log (Treated sample)

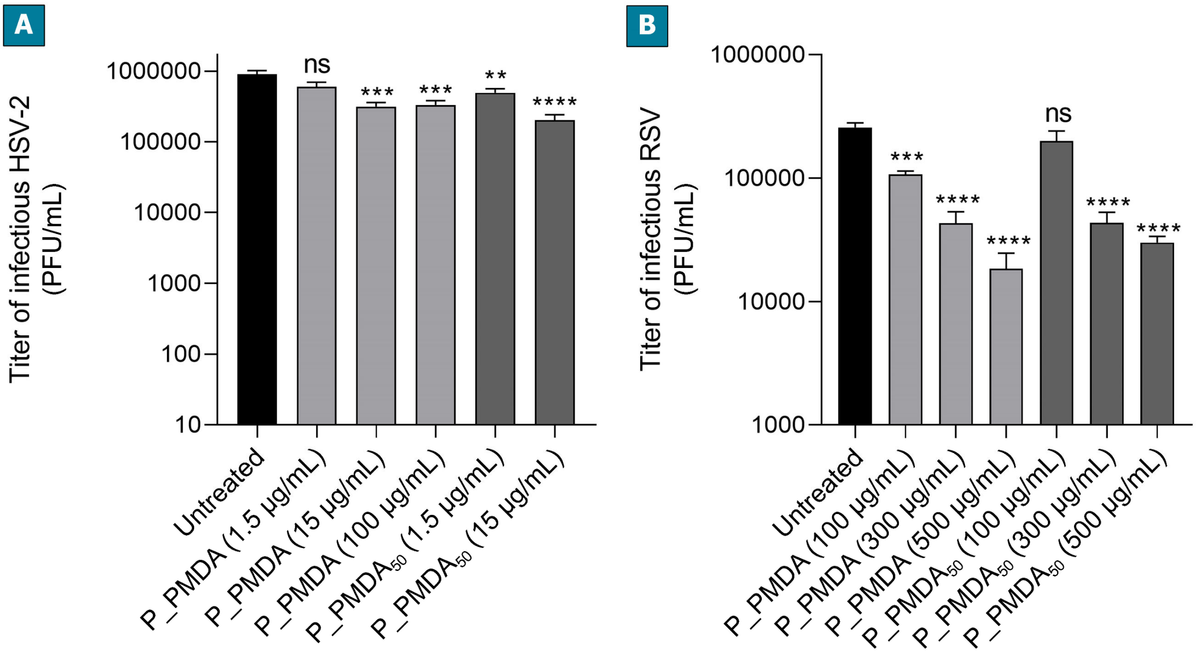

Figure S8. HSV-2 and RSV inactivation assays with additional concentrations of compounds. HSV-2 (A) and RSV (B) inactivation assays. HSV-2 or RSV infectious particles were additionally incubated with 100 µg/mL of P_PMDA or P_PMDA$_{50}$ for 2 hours at 37°C and the residual viral infectivity was than evaluated by titration to the non-inhibitory concentration of the polymers. Graphs including all tested doses are reported. On the y-axis, the infectious titers are expressed as plaque-forming units per mL (PFU/mL). Error bars represent standard error of the mean (SEM) of three independent experiments (ANOVA and Bonferroni Post hoc test; ns p>0.05, **p<0.005, ***p<0.0005, ****p<0.0001

Table S2. Raw data of P_PMDA and P_PMDA$_{50}$ binding inhibition activities

	Sample	Titer (PFU/mL)	*R
**RSV**	Untreated	7119	
	P_PMDA (500 μg/mL)	2655	0.4
	P_PMDA (300 μg/mL)	1508	0.7
	P_PMDA$_{50}$ (500 μg/mL)	1917	0.6
	P_PMDA$_{50}$ (300 μg/mL)	1755	0.6
**HSV-2**	Untreated	251809	
	P_PMDA (1.5 μg/mL)	7721	1.5
	P_PMDA (15 μg/mL)	2817	2.0
	P_PMDA (100 μg/mL)	5428	1.7
	P_PMDA$_{50}$ (1.5 μg/mL)	68876	0.6
	P_PMDA$_{50}$ (15 μg/mL)	13355	1.3
	P_PMDA$_{50}$ (100 μg/mL)	25158	1.0
	P_PMDA$_{50}$ (300 μg/mL)	5802	1.8
	P_PMDA$_{50}$ (500 μg/mL)	6056	1.8

***Logarithm of inhibition R** = Log (Untreated sample) – Log (Treated sample)

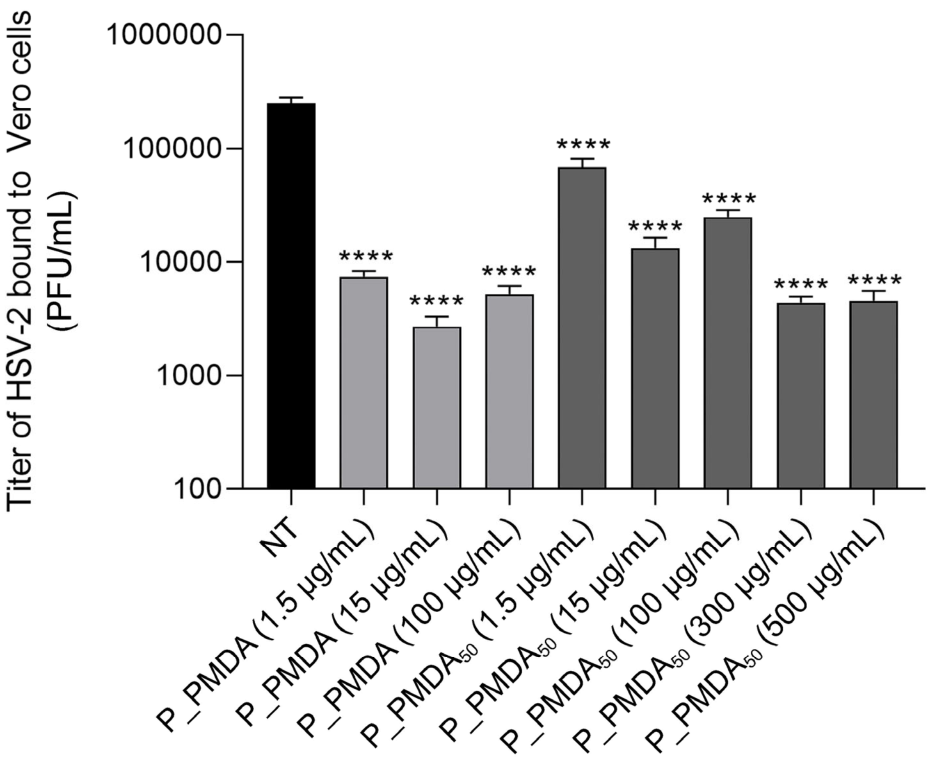

Figure S9. HSV-2 and RSV binding assays with additional concentrations of compounds. HSV-2 (MOIs = 3) was allowed to attach to cells in presence of additional 100 μg/mL of P_PMDA, or 100, 300 and 500 μg/mL of P_PMDA. The cell-bound virus titers were determined by means of titration on confluent cells. A graph including all tested doses is reported. On the y-axis, the infectious titers are expressed as plaque-forming units per mL (PFU/mL). Error bars represent standard error of the mean (SEM) of three independent experiments (ANOVA and Bonferroni Post hoc test; ****p<0.0001).
